# Effect of exercise training on blood pressure variability in adults: A systematic review and meta-analysis

**DOI:** 10.1371/journal.pone.0292020

**Published:** 2023-10-18

**Authors:** Min Lin, Yipin Lin, Yuhua Li, Xiongbiao Lin

**Affiliations:** 1 Department of Electro-Cardiographic Information, The First Affiliated Hospital of Xiamen University, Xiamen, China; 2 Xiamen Key Laboratory of Cardiac Electrophysiology, Xiamen, China; 3 The Shengli Clinical College, Fujian Medical University, Fuzhou, China; Universiti Malaya, MALAYSIA

## Abstract

**Background and aims:**

Targeting blood pressure variability (BPV) can potentially reduce cardiovascular events and incidence of mortality, but whether exercise reduces BPV remains controversial. This systematic review and meta-analysis were designed to study the impact of an exercise intervention on BPV in adults.

**Methods:**

A systematic search of PubMed, Web of Science, Scopus, EBSCO host, Cochrane, Embase, Science direct databases was done to retrieve controlled trials published from inception to January 10, 2023 that investigated the effects of exercise on BPV. The main characteristics of each study were synthesized, re-evaluated, and used in this meta-analysis.

**Results:**

Eleven studies with 514 adults with exercise training were eligible for single-arm meta-analysis and six randomized controlled trials (RCTs) were selected for further meta-analysis. After exercise training, systolic blood pressure variability (SBPV) (effect size = -0.76, 95%CI: -1.21 to -0.30, *I*^*2*^ 60%), especially the average real variability SBP (-0.85, -1.44 to -0.27, *I*^*2*^ 59%), was significantly improved. SBPV (-0.68, –1.18 to -0.18, *I*^*2*^ 64%) significantly improved in hypertension patients. Aerobic exercise improved SBPV (-0.66, -1.32 to -0.00, *I*^*2*^ 45%), and combined training improved both SBPV (-0.74, -1.35 to -0.14, *I*^*2*^ 65%) and diastolic blood pressure variability (DBPV) (-0.36, -0.65 to -0.02, *I*^*2*^ 33%). The SBPV of daytime (-0.90, -1.39 to -0.40, *I*^*2*^ 57%) and DBPV of daytime (-0.31, -0.53 to -0.08, *I*^*2*^ 0%) values demonstrated significant improvement compared to the night-time values. Moreover, six RCTs demonstrated a decrease in SBPV (-1.03, -1.77 to -0.28, *I*^*2*^ 45%).

**Conclusion:**

This study provides quantitative evidence that exercise training can improve BPV, especially SBPV, in adults. This meta-analysis suggests that aerobic exercise and combined training should be recommended for hypertension patients.

## Introduction

Premature death has been strongly associated with hypertension, which has emerged as a strong predictor of cardiovascular disease and the onset of severe life-threatening cardiovascular events [1]. For decades, accumulating evidence has indicated that increasing blood pressure variability (BPV) is associated with a higher likelihood of excessive blood pressure (BP) fluctuations, leading to various high-flow cardiovascular events and organ damage, including to the brain [2,3]. Thus, targeting large changes in BPV may be a useful solution to prevent cerebral small vessel disease and reduce the risk of stroke and dementia [3].

BPV is defined as continuous dynamic fluctuations in BP over a period of time. There are several types of BPV: very short-term (beat-to-beat), short-term (within 24 h), mid-term (day-to-day), and long-term (between clinic visits over months and years) [2]. Ambulatory blood pressure monitoring (ABPM) is an efficient diagnostic tool that can distinguish between BPV types. ABPM is advantageous because it is portable, non-invasive, free of painless characteristics, and allows for accurate measurements of BPV over the course of a day, including all daily activities, work and sleep [4]. The complex underlying physiology of BPV relies on interactions between hemodynamic, neuronal, humoral, behavioral (anxiety, postural changes, and lifestyle), and environmental factors [5].

Antihypertensive exercise has been widely recognized as an effective therapy to reduce BP. Lowering BP through regular exercise rather than medication is more acceptable for younger patients with early-onset hypertension. To achieve optimal health outcomes, the World Health Organization advises 150–300 min of moderate intensity, 75–150 min of high intensity, or an equivalent combination of aerobic physical activity per week for adults [6]. However, whether exercise intervention is an effective way to reduce BPV remains a controversial issue. Evaluating the current evidence regarding exercise intervention and BPV may inform BP control programs. Therefore, we designed a systematic review and meta-analysis to investigate the effectiveness of exercise intervention on BPV in adults.

## Methods

A systematic review and meta-analysis was conducted and reported according to the recommendations of the Preferred Reporting Items for Systematic Literature Review (PRISMA) [7] (S2 File). The protocol was registered with the International Prospective Register of Systematic Reviews (CRD42023399829).

### Search strategy

All studies reporting the effect of exercise training on BPV from ABPM in adults were reviewed without any limitations regarding year of publication or language. An extensive search of the published literature from inception to January 10, 2023 was done using PubMed, Web of Science, Scopus, EBSCOhost, Cochrane, Embase, Science direct databases and transferred to information management software (Endnote 20, America). The literature search was applied to identify peer-reviewed original research articles using keyword MeSH headings as follows: (exercise OR physical activity) AND (“blood pressure variability” OR BPV). The details of the strategy are presented in (S1 File). A “reference search” of the retrieved articles, a "cited reference search" in the Cochrane Collaboration database, and a review of author’s files were also performed.

### Eligibility criteria

The following inclusion criteria were applied to select articles: 1) clinical trial: including single-arm clinical trial or randomized controlled trial (RCT); 2) studies that reported BPV data from 24h-ABPM before and after exercise training, with or without a control group (no exercise training); and 3) exercise interventions that were administered for at least 3 weeks. Studies with the following were excluded: 1) case reports, conference abstracts, reviews, meta-analysis or guidelines; 2) animal studies; 3) combined with other interventions (such as dietary or medicine); 4) did not report coefficient of variability (CV) and average real variability (ARV) for 24h-BPV; 5) studies from the same clinical trial; and 6) non-adult subjects.

### Study selection and data extraction

The titles and abstracts of articles were all reviewed by two independent reviewers (Min Lin and Yipin Lin) using clear inclusion and exclusion criteria. The full text of potentially relevant articles was then retrieved for full review. Any discrepancies regarding study eligibility were decided by a third reviewer (Yuhua, Li). Two reviewers (Min Lin and Yipin Lin) collected data from each report independently, and a third reviewer (Yuhua, Li) confirmed the data extraction. Data were finally reviewed and obtained as follows: authors, publication year, country, study objective, study design, inclusion and exclusion criteria, sample size, hypertension history, average age, sex, exercise type and intensity, periods of intervention, and outcomes of BPV.

#### Outcome measurements

The outcomes are presented as CV and ARV. We excluded the standard deviation (SD) of BP because it is affected by absolute BP. We followed each step of the protocol meticulously, and if there was any deviation from the designed study, the study was not approved by the committee. All parameters of BPV were defined as follows:

SBPV: systolic blood pressure variability in 24 h
DBPV: diastolic blood pressure variability in 24 h
SBPVd/ SBPVn: systolic blood pressure variability in day-time or night-time
DBPVd/ DBPVn: diastolic blood pressure variability in day-time or night-time

$SD=\sqrt{\frac{1}{n}\sum_{i=0}^{n-1}\left[{BP}_{i+1}-{BP}_{mean}\right]}2$

CV = SD/BP_mean_

$ARV=\frac{1}{n-1}\sum_{i=0}^{n-1}|{BP}_{i+1}-{BP}_{i}|$

### Quality assessment

Reviewers assessed the quality of the included study articles using the Scottish Intercollegiate Guidelines Network (SIGN) criteria. The evaluation report of each reviewer was calculated and the significant aspect essential for the study was highlighted. The evaluation included 10 items of bias for RCTs and seven for single-arm clinical trials. Each item had four answers, including “yes”, “no”, “can’t say”, or “not applicable” [7] (S3 File).

### Statistical considerations

*P*-values <0.05 were considered statistically significant among the studies included in our meta-analysis. The main characteristics of each study are presented, and continuous variables were extracted as mean ± SD using reviewer Manager 5.4. First, we calculated the effect size of each BPV parameter after exercise and compared the value with baseline (before exercise). Then we calculated the effect size of BPV changes after exercise between the exercise group and control group. A negative effect size indicated a reduced BPV after exercise. A positive effect size indicated an increased BPV after exercise. Funnel plots were used for publication bias assessment and *I*^2^ values were used to examine study heterogeneity. If the *I*^2^ value was significant (*I*^2^>50%), the random effect model was used for statistical analysis. If the study was homogeneous (*I*^2^<50%), the fixed effect model was used. Subgroup analysis was done to explore the causes of heterogeneity and sensitivity by excluding one single study at a time in sequence.

## Results

### Study selection

**Fig 1:**
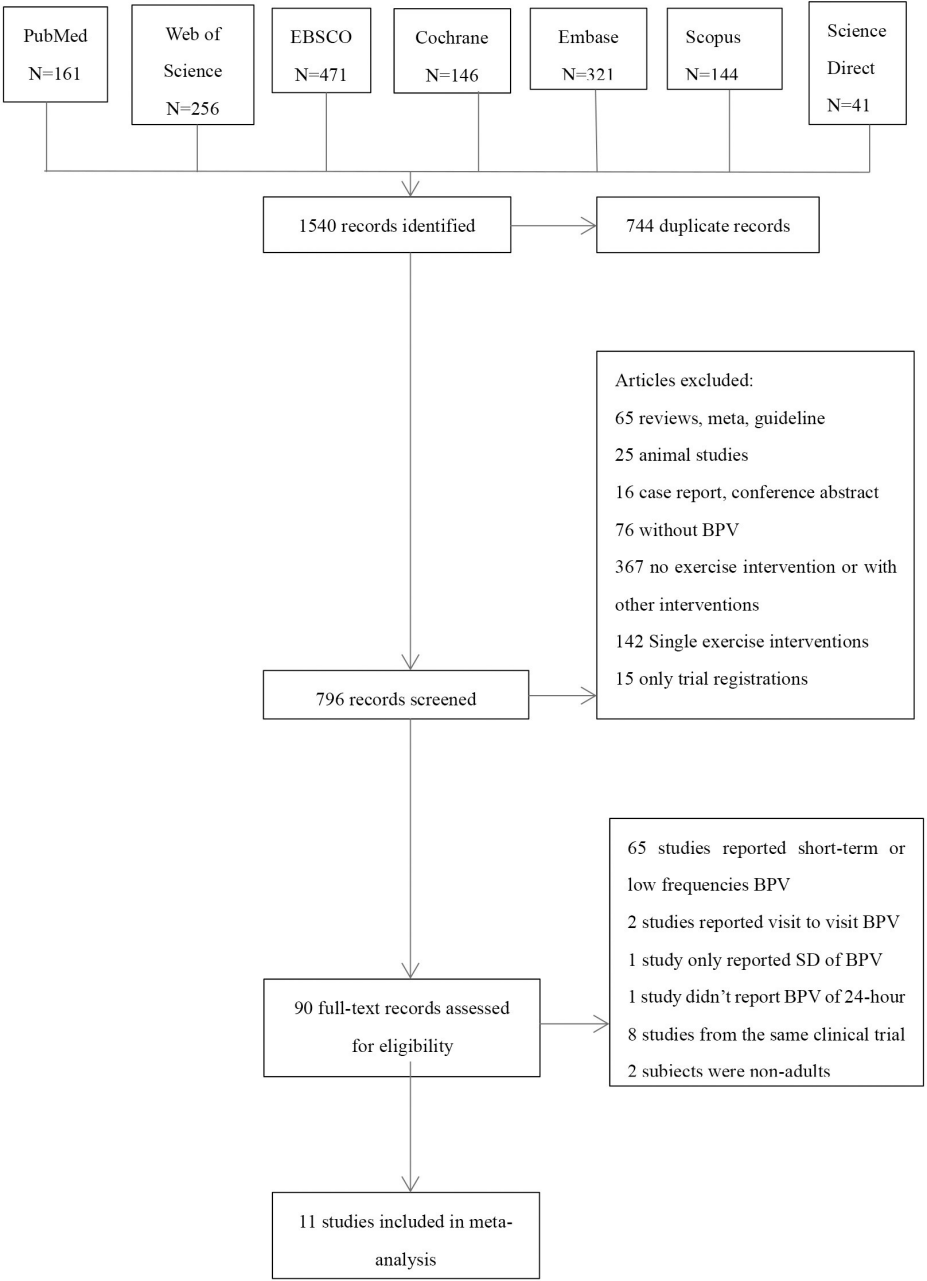
Flow diagram in accordance with PRISMA guideline.

A total of 1540 articles were initially retrieved. The detailed screening process is provided in Fig 1. The main characteristics and outcomes of the final 11 included studies (*n* = 694) are reported in Table 1. We compared changes in BPV before and after exercise training in 514 adults. Among them, six RCTs that compared exercise groups and no-exercise groups were selected for further analysis.

**Table 1:** Characteristics of included studies.

Study							Country	Study design	Patient				Exercise						Outcome
									HT or NT	Group (n)	Age	Male n(%)	Exercise type	Duration /Session	Intensity	Session /Week	Week	Supervision	
Diaz, 2012							America	SACT	HT	Exercise (14)	NA	NA	Aerobic	40min	65% of maximum oxygen uptake	3	24	Yes	CV, SD, ARV, ASV
Katrina, 2019							UK	RCT	HT	Exercise (24) Control (24)	43.8±7.3	NA	Isometric	14min	Mean knee joint training angle was 148°±19°	3	4	No	ARV
Mariano, 2020							Brazil	SACT	HT NT	Exercise (13) Exercise (13)	52.7 ± 5.3 58.9 ± 3.9	0(0)	Combined	45min	60% of maximal between ventilatory thresholds 1 and 2	3	10	Yes	SD ARV
Betolaza, 2020							Spain	RCT	HT	Exercise (179) Control (52)	54.2 ± 7.2 53.1 ± 8.6 54.4 ± 7.2 52.9 ± 8.5	38(63.3) 40(65.6) 41(66.1) 33(55.9)	MICT HVHIIT LVHIIT	20-45min	individual heart rate responses	2	16	Yes	SD,CV
Caminiti, 2021							Italy	SACT	HT	Exercise (55)	67.6 ± 6.6 69.0± 10.4	55(100)	Aerobic; combined	80min	Rating of perceived exertion of 13–14; 60% of 1-RM	3	12	No	ARV
Chehuen, 2021							Brazil	RCT	Peripheral vascular disease	Exercise (19) Control (16)	63.0± 7.0 62.0± 7.0	35(100)	Walk	30min	At pain threshold	2	12	Yes	SD,ARV
Jamie, 2021							UK	RCT	NT	Exercise (21) Control (20)	22.8±2.7	20(48.78)	HIIT	8.5min	Maximum effort	3	4	Yes	ARV
Seidel, 2021							Germany	RCT	HT	Exercise (43) Control (23)	60.7±9.9	25(60.98)	Aerobic; handgrip; sham	30min 12min	Borg scale 12–13; 30% maximal power	5	12	Yes	CV
**Baross,** **2022**	8.99	1.67	13	10.84	2.17	13	UK	RCT	NT	Exercise (13) Control (12)	23.0 ± 4.0	16(64)	Isometric resistance training	30-45min	Moderate	2–4	8	Yes	ARV
Batista, 2022							Brazil	SACT	HT	Exercise (23)	58.0±5.0	23(100)	Mat Pilates	50min	Borg’s rate 11–15	3	12	Yes	SD,ARV
Caminiti, 2022							Italy	SACT	HT	Exercise (64)	66.1± 12.7	46(71.8)	Combined	60min	Rating of perceived exertion 13–14 50–60% 1-RM	3	12	Yes	ARV

SACT: Single-arm clinical trial; RCT: Randomized controlled trial; HT: Hypertension; NT: Non-hypertension; NA: None; CV: Coefficient of variability; ARV: Average real variability; SD: Standard deviation of blood pressure; MICT: Moderate-intensity continuous training; HVHIIT: High-volume and high-intensity interval training; LVHIIT: Low-volume and high-intensity interval training.

### Quality of articles and sensitivity analyses

The answer “yes” of SIGN varied from 40% to 80%, with a mean score of 55.83±12.40% in 11 studies. In six RCTs, the results with “yes” varied from 70% to 80%, with a higher mean score of 61.43±3.73% (S4 File).

### Characteristics of population and intervention

#### Sample size

We included a total of 694 adults: 514 adults had exercise training in the 11 studies, and 180 adults were in the control group without exercise training. The sample size ranged from 14 to 238 adults in one study.

#### Research objective

Seven studies [8–14] included hypertension patients, two studies [15,16] included healthy adults, and one study [17] compared both. The other study^18^ focused on patients with peripheral vascular disease.

#### Age

The mean age of adults in the exercise groups ranged from 22.8±2.7 years to 69.0±10.4 years, and two studies [15,16] included young adults.

#### Gender

The proportion of male adults varied from 0% to 100%.

#### Exercise type

Four studies [8,11,12,18] conducted aerobic training, three studies [9,12,16] provided resistance training, and four studies [10,11,14,17] analyzed combined aerobic and resistance training. Two studies [10,15] included HIIT intervention. One study [13] used Mat Pilates.

#### Exercise duration and frequency

The duration of each exercise session ranged from 8.5 min to 60 min in all studies, and exercise training gradually increased in two studies. Maintenance of exercise frequency ranged from 2 to 5 times per week, and most studies (7/11) included a frequency of 3 times per week. The duration of exercise intervention varied from 1 to 6 months.

#### Exercise intensity

Diaz, et al [8] included a 65% of VO_2_ max; Katrina, et al [9] used mean knee joint training angle 148°±19°; Seidel, et al [12] used 30% maximal power; two studies [16,17] selected 50%-60% of maximal between ventilatory thresholds; Betolaza, et al [10] used individual heart rate responses; four studies [11–14] presented the Borg scale [11–15]; Chehuen, et al [18] used a pain threshold; and Jamie, et al [15] used maximum effort.

#### Supervised exercise

Almost all studies included supervised exercise training; only two studies [9,11] had no supervised exercise training.

## Meta-analysis

### Single-arm meta-analysis

**Fig 2:**
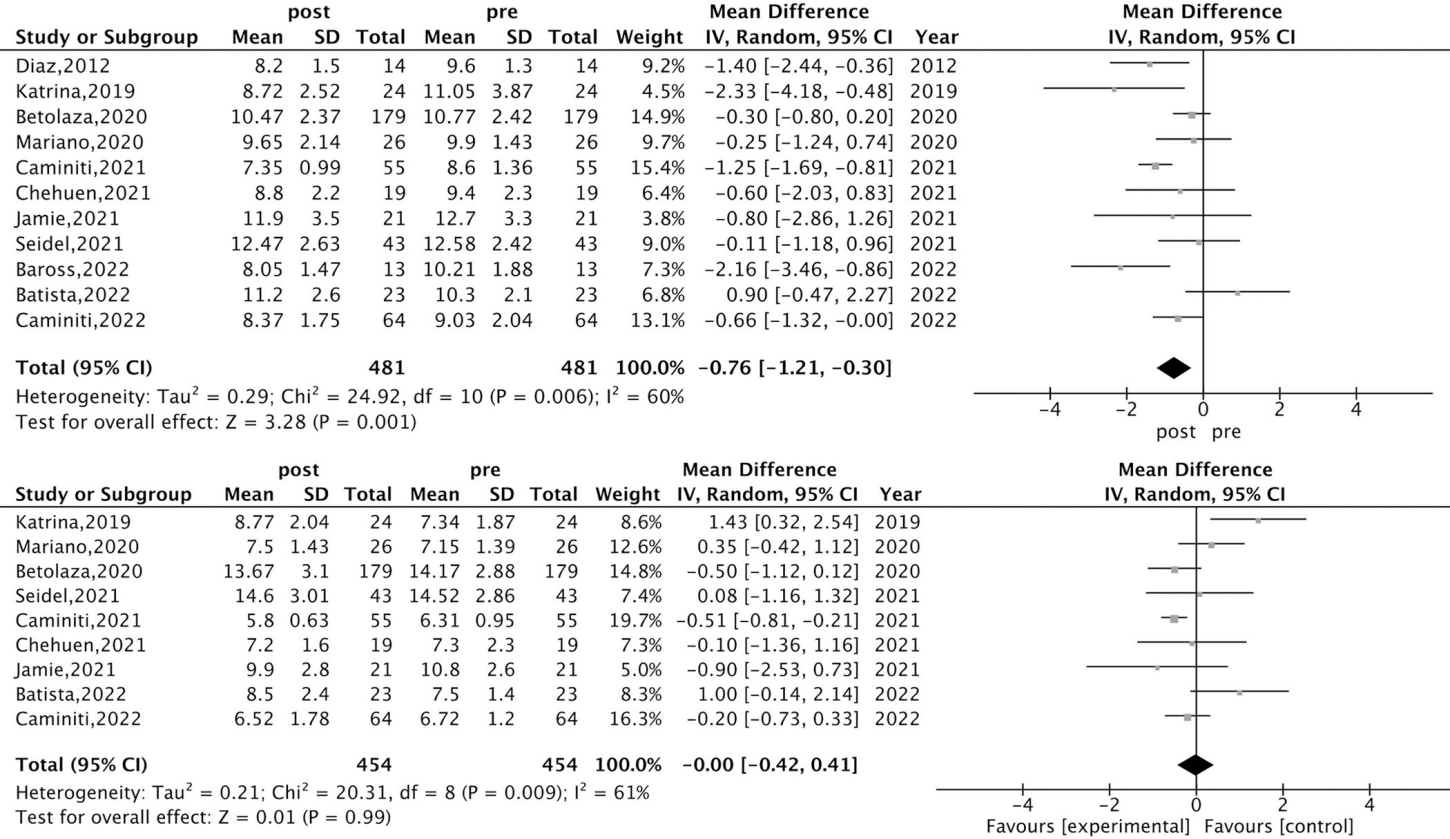
Meta-analysis of exercise effects on SBPV and DBPV. The results are presented as mean difference and 95% confidence interval in forest plots.

Compared with pre-exercise training, the SBPV_24h_ significantly improved in adults after exercise training (effect size = -0.76, 95%CI -1.21 to -0.30, *I*^*2*^ 60%), but the DBPV (-0.00, -0.42 to 0.41, *I*^*2*^ 61%) did not significantly change (Fig 2).

### Subgroup analysis

**Fig 3:**
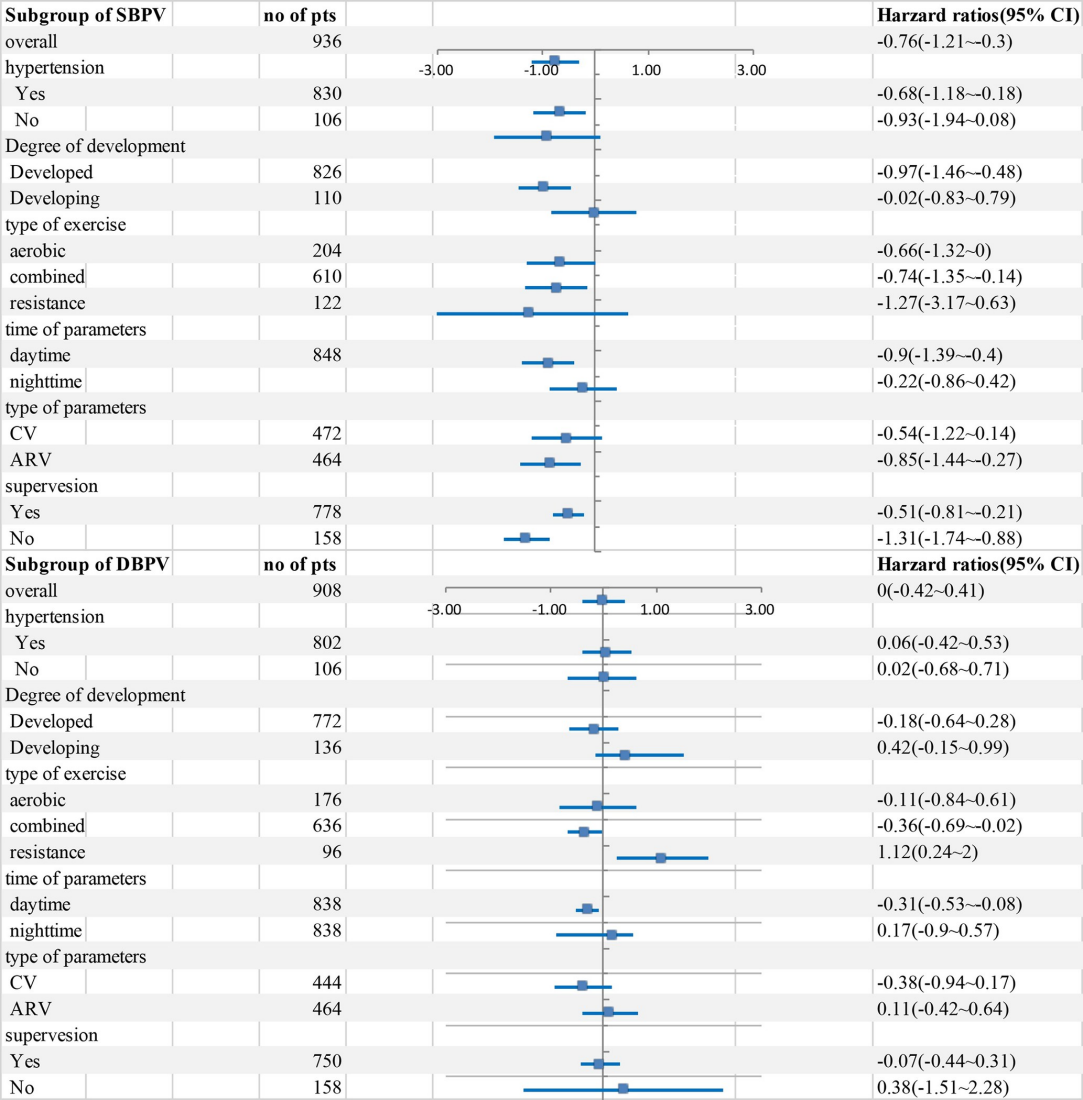
Subgroup analysis of SBPV and DBPV. Subgroup analysis was done for hypertension, degree of development, type of exercise, time of parameters, type of parameters, and supervision.

After aerobic exercise training, SBPV (-0.66, -1.32 to -0.00, *I*^*2*^ 45%) improved, but DBPV (-0.11, -0.84 to 0.61, *I*^*2*^ 50%) did not change. In subgroup analysis of combined training, both SBPV (-0.74, -1.35 to -0.14, *I*^*2*^ 65%) and DBPV (-0.36, -0.65 to -0.02, *I*^*2*^ 33%) improved. For resistance exercise, SBPV had uncertain benefits (-1.27, -3.17 to 0.63, *I*^*2*^ 79%), and DBPV (1.12, 0.24 to 2.00, *I*^*2*^ 0%) increased. For hypertension patients, the SBPV (-0.68, -1.18 to -0.18, *I*^*2*^ 64%) displayed significant improvement, but among healthy adults, the SBPV (-0.93, -1.94 to 0.08, *I*^*2*^ 44%) did not significantly change. There were no obvious changes in DBPV in either hypertension patients (0.06, -0.42 to 0.53, *I*^*2*^ 67%) or healthy adults (0.02, -0.68 to 0.71, *I*^*2*^ 0%). SBPV (-0.97, -1.46 to -0.48, *I*^*2*^ 60%) improved in adults in developed countries. However, the DBPV (-0.18, -0.64 to 0.28, *I*^*2*^ 60%) in developed countries and the SBPV (-0.02, -0.83 to 0.79, *I*^*2*^ 0%) and DBPV (0.42, -0.15 to 0.99, *I*^*2*^ 0%) in developing countries were not significantly different. The SBPV_d_ (-0.90, -1.39 to -0.40, *I*^*2*^ 57%) and DBPV_d_ (-0.31, -0.53 to -0.08, *I*^*2*^ 0%) were significantly improved compared with SBPV_n_ (-0.22, -0.86 to 0.42, *I*^*2*^ 56%) and DBPV_n_ (0.17, -0.90 to 0.57, *I*^*2*^ 64%). The ARV_SBP_ (-0.85, -1.44 to -0.27, *I*^*2*^ 59%) declined after exercise training, but CV_SBP_ (-0.54, -1.22 to 0.14, *I*^*2*^ 49%), CV_DBP_ (-0.38, -0.94 to 0.17, *I*^*2*^ 0%), and ARV_DBP_ (0.11, -0.42 to 0.64, *I*^*2*^ 69%) did not change. SBPV declined after exercise training in the supervision group (-0.51, -0.81 to -0.21, *I*^*2*^ 46%) and non-supervision group (-1.31, -1.74 to -0.88, *I*^*2*^ 19%). There were no changes in DBPV in either the supervision group (-0.07, -0.44 to 0.31, *I*^*2*^ 22%) or non-supervision group (0.38, -1.51 to 2.28, *I*^*2*^ 91%) (Fig 3).

### Meta-analysis of RCTs

**Fig 4:**
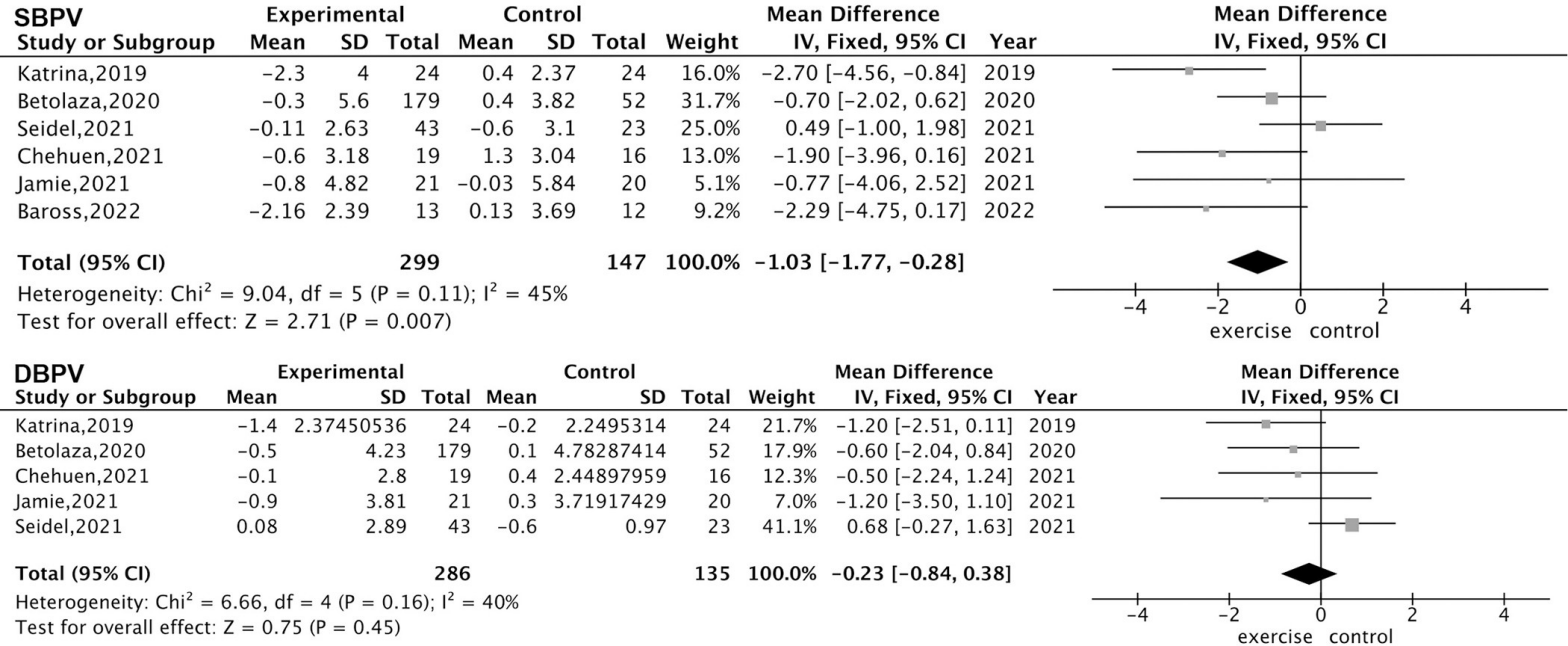
Meta-analysis of exercise on SBPV and DBPV in RCTs. Six RCTs were selected for high proof level of analysis and are presented as forest plots with mean difference and 95% confidence intervals.

A total of six RCTs with the highest level of proof demonstrated a decrease in SBPV (-1.03, -1.77 to -0.28, *I*^*2*^ 45%), but no significant change in DBPV (-0.23, -0.84 to 0.38, *I*^*2*^ 40%) (Fig 4). For different parameters of BPV, ARV_SBP_ (-2.14, -3.28 to -1.01, *I*^*2*^ 0%) and ARV_DBP_ (-0.99, -1.94 to -0.04, *I*^*2*^ 0%) both improved with exercise. There were no changes in CV_SBP_ (-0.18, -1.17 to 0.81, *I*^*2*^ 27%) and CV_DBP_ (0.42, -0.41 to 1.24, *I*^*2*^ 17%) between the exercise group and control group.

## Publication bias and sensitivity analysis

No significant deviation was observed in the funnel plots of our meta-analysis (S5 File). There was little influence on effect size after excluding one study at a time in sequence.

## Discussion

In this meta-analysis, we found that exercise training improved BPV parameters, especially SBPV, in adults with a high level of proof. BPV in the daytime and ARV resulted in significant reductions. Thus, BPV in the daytime and ARV could be accurate parameters to reflect the effect of exercise. Aerobic exercise and combined training were the recommended exercise types in this meta-analysis, and whether the exercise was supervised or not had little influence on BPV. To our knowledge, this is the first meta-analysis of RCTs that assessed the effects of exercise training on BPV.

Hypertension is a major risk factor for brain damage. Proper management of hypertension and BPV is beneficial to maintain optimal cognitive function, as regulating BPV can reduce the risk of vascular dementia and the global burden of stroke [19]. More specifically, controlling regional cerebral blood flow to the brain’s white matter can protect cognition. Thus, improving BP and BPV may be an intermediate process to slow organ damage and mortality. Regular physical activity can reduce the risk of all-cause mortality and several chronic medical conditions [20]. Previous studies have provided evidence that exercise training has an overall beneficial effect on BP. Our meta-analysis showed that aerobic and combination exercise was a cost-effective approach to improve BPV. Our meta-analysis showed that adults with hypertension achieved BPV reduction through exercise. Different types of exercise may results in different effects. Isometric handgrip exercise had uncertain efficacy in hypertension patients. Therefore, handgrip exercise was not included in the current guidelines for the treatment of hypertension [21], as our subgroup meta-analysis showed resistance training had no benefits on BPV, and even increased DBPV. Guidelines [6] recommend sufficient duration and moderate or high intensity exercise to improve cardiovascular outcomes. Our results also support the efficacy of aerobic and combined exercise on both SBPV and DBPV. Thus, combined exercise regimens may be recommended to reduce BPV. However, the increase of resistance exercise on DBPV demonstrated that solitary resistance exercise can also reduce BPV.

The PURE study showed that physical activity levels increased with reported increased incomes, but this trend was not significant [22]. People in developed countries may have a higher degree of recognition of the importance of exercise. Advocacy and guidance in developing countries may also be important factors. As people become more aware of the benefits of exercise, the need for supervision becomes less important. Both supervised and unsupervised subgroups performed well in reducing BPV in this meta-analysis. This is important because unsupervised exercise is more cost-effective to promote.

The mechanism underlying the association between BPV and cardiovascular events is not clear [23]. Short term variability of BP is affected by cardiovascular physiology and cardiac rhythm that can be determined by changes in behavior, emotion, and posture [24]. Increased oxidative stress that occurs in hypertension can lead to endothelium-dependent vasodilation, but prolonged high-intensity endurance exercise may improve responses to oxidative stress [25]. Thus, moderate exercise intensity and frequency should be considered to reduce BPV. Exercise may change resting BP by modulating levels of catecholamines [26], such as angiotensin II and nitric oxide, which are potential mediators that can improve vagal tone [27]. Future studies should explore the mechanisms that underly the benefits of exercise training on BPV.

There are some limitations of our study that should be noted. First, the lack of consensus on BPV measurement and quantification may have led to heterogeneity across the studies in our analysis. However, in our review we selected two relatively stable and independent parameters (CV and ARV) for subgroup analysis. Secondly, differences in exercise type, intensity, and duration may have also affected study heterogeneity. All included studies were clinic trails, but some of the studies had no control group (without exercise intervention). Lastly, only six RCTs were included in this meta-analysis, the number of subjects was small, and the representativeness was also limited. Future analysis should include a larger sample size of related RCTs.

## Conclusion

Exercise training improved BPV, especially SBPV, in adults, and the level of proof was high. BPV in the daytime and the parameter of ARV resulted in significant reduction in BPV. Aerobic exercise and combined training were the most recommended exercise types across our analysis.

## Supporting information

S1 Checklist PRISMA 2020 checklist. (DOCX)

S1 File Search strategy. (PDF)

S2 File PRISM checklist. (PDF)

S3 File SIGN assessment. (PDF)

S4 File SIGN scores of 11 selected studies. (PDF)

S5 File Funnel plot. (TIF)
