# Supplementary material for: Effect of exercise training on blood pressure variability in adults: A systematic review and meta-analysis
Source: PLoS One. 2023 Oct 18;18(10):e0292020. doi: 10.1371/journal.pone.0292020 (PMC10584136; doi:10.1371/journal.pone.0292020)
Supplement: S1 File — (PDF) [file pone.0292020.s002.pdf]

Search strategy

Pubmed

((((((((((((((((((((((((((exercise[MeSH Terms]) OR (Exercises[Title/Abstract])) OR (Physical Activity[Title/Abstract])) OR (Activities, Physical[Title/Abstract])) OR (Activity, Physical[Title/Abstract])) OR (Physical Activities[Title/Abstract])) OR (Exercise, Physical[Title/Abstract])) OR (Exercises, Physical[Title/Abstract])) OR (Physical Exercise[Title/Abstract])) OR (Physical Exercises[Title/Abstract])) OR (Acute Exercise[Title/Abstract])) OR (Acute Exercises[Title/Abstract])) OR (Exercise, Acute[Title/Abstract])) OR (Exercises, Acute[Title/Abstract])) OR (Exercise, Isometric[Title/Abstract])) OR (Exercises, Isometric[Title/Abstract])) OR (Isometric Exercises[Title/Abstract])) OR (Isometric Exercise[Title/Abstract])) OR (Exercise, Aerobic[Title/Abstract])) OR (Aerobic Exercise[Title/Abstract])) OR (Aerobic Exercises[Title/Abstract])) OR (Exercises, Aerobic[Title/Abstract])) OR (Exercise Training[Title/Abstract])) OR (Exercise Trainings[Title/Abstract])) OR (Training, Exercise[Title/Abstract])) OR (Trainings, Exercise[Title/Abstract]))）) AND ((((((((\"blood pressure variability\"[Title/Abstract]) OR (\"variable blood pressure\"[Title/Abstract])) OR (\"BPV\"[Title/Abstract])) OR (\"varia* in blood pressure\"[Title/Abstract])) OR (\"average real variability\"[Title/Abstract])) OR (\"variance independent of the mean\"[Title/Abstract])) OR (\"blood pressure fluctuat*\"[Title/Abstract]))）

A total of 161 articles

Web of science

（TS= (exercise）OR AB= (Exercises OR Physical Activity OR Activities, Physical OR Activity, Physical OR Physical Activities OR Exercise, Physical OR Exercises, Physical OR Physical Exercise OR Physical Exercises OR Acute Exercise OR Acute Exercises OR Exercise, Acute OR Exercises, Acute OR Exercise, Isometric OR Exercises, Isometric OR Isometric Exercises OR Isometric Exercise OR Exercise, Aerobic OR Aerobic Exercise OR Aerobic Exercises OR Exercises, Aerobic OR Exercise Training OR Exercise Trainings OR Training, Exercise OR Trainings, Exercise)) AND (AB=(\"blood pressure variability\" OR \"variable blood pressure\" OR \"BPV\" OR \"varia* in blood pressure\" OR \"average real variability\" OR \"variance independent of the mean\" OR \"blood pressure fluctuat*\")）

A total of 256 articles

EBSCOhost

S1 AB "blood pressure variability" OR "variable blood pressure" OR "BPV" OR "varia in blood pressure" OR "average real variability" OR "variance independent of the mean" OR "blood pressure fluctuat*"*

S2 SU exercise OR AB (Exercises OR Physical Activity OR Activities, Physical OR Activity, Physical OR Physical Activities OR Exercise, Physical OR Exercises, Physical OR Physical Exercise OR Physical Exercises OR Acute Exercise OR Acute Exercises OR Exercise, Acute OR Exercises, Acute OR Exercise, Isometric OR Exercises, Isometric OR Isometric Exercises OR Isometric Exercise OR Exercise, Aerobic OR Aerobic Exercise OR Aerobic Exercises OR Exercises, Aerobic OR Exercise Training OR Exercise Trainings OR Training, Exercise OR Trainings, Exercise)

S3 (SU exercise OR AB Exercises OR Physical Activity OR Activities, Physical OR Activity, Physical OR Physical Activities OR Exercise, Physical OR Exercises, Physical OR Physical Exercise OR Physical Exercises OR Acute Exercise OR Acute Exercises OR Exercise, Acute OR Exercises, Acute OR Exercise, Isometric OR Exercises, Isometric OR Isometric Exercises OR Isometric Exercise OR Exercise, Aerobic OR Aerobic Exercise OR Aerobic Exercises OR Exercises, Aerobic OR Exercise Training OR Exercise Trainings OR Training, Exercise OR Trainings, Exercise) AND (S1 AND S2)

A total of 471 articles

Cochrane

exercise OR Exercises OR Physical Activity OR Activities, Physical OR Activity, Physical OR Physical Activities OR Exercise, Physical OR Exercises, Physical OR Physical Exercise OR Physical Exercises OR Acute Exercise OR Acute Exercises OR Exercise, Acute OR Exercises, Acute OR Exercise, Isometric OR Exercises, Isometric OR Isometric Exercises OR Isometric Exercise OR Exercise, Aerobic OR Aerobic Exercise OR Aerobic Exercises OR Exercises, Aerobic OR Exercise Training OR Exercise Trainings OR Training, Exercise OR Trainings, Exercise AND "blood pressure variability" OR "variable blood pressure" OR "BPV" OR "varia* in blood

pressure" OR "average real variability" OR "variance independent of the mean" OR "blood pressure fluctuat*"

A total of 146 articles

Embase

('blood pressure variability':ab,ti OR 'variable blood pressure':ab,ti OR 'bpv':ab,ti OR 'varia* in blood pressure':ab,ti OR 'average real variability':ab,ti OR 'variance independent of the mean':ab,ti OR 'blood pressure fluctuat*':ab,ti) AND (exercise:ab,ti OR exercises:ab,ti OR 'physical activity':ab,ti OR 'activities, physical':ab,ti OR 'activity, physical':ab,ti OR 'physical activities':ab,ti OR 'exercise, physical':ab,ti OR 'exercises, physical':ab,ti OR 'physical exercise':ab,ti OR 'physical exercises':ab,ti OR 'acute exercise':ab,ti OR 'acute exercises':ab,ti OR 'exercise, acute':ab,ti OR 'exercises, acute':ab,ti OR 'exercise, isometric':ab,ti OR 'exercises, isometric':ab,ti OR 'isometric exercises':ab,ti OR 'isometric exercise':ab,ti OR 'exercise, aerobic':ab,ti OR 'aerobic exercise':ab,ti OR 'aerobic exercises':ab,ti OR 'exercises, aerobic':ab,ti OR 'exercise training':ab,ti OR 'exercise trainings':ab,ti OR 'training, exercise':ab,ti OR 'trainings, exercise':ab,ti)

A total of 321 articles

Scopus

ALL (exercise OR exercises OR physical AND activity OR activities, AND physical OR activity, AND physical OR physical AND activities OR exercise, AND physical OR exercises, AND physical OR physical AND exercise OR physical AND exercises OR acute AND exercise OR acute AND exercises OR exercise, AND acute OR exercises, AND acute OR exercise, AND isometric OR exercises, AND isometric OR isometric AND exercises OR isometric AND exercise OR exercise, AND aerobic OR aerobic AND exercise OR aerobic AND exercises OR exercises, AND aerobic OR exercise AND training OR exercise AND trainings OR training, AND exercise OR trainings, AND exercise AND "blood pressure variability" OR "variable blood pressure" OR "BPV" OR "varia* in blood pressure" OR "average real variability" OR "variance independent of the mean" OR "blood pressure fluctuat*")

A total of 144 articles

Science Direct

("blood pressure variability" OR “BPV”) AND (“exercise intervention” OR “exercises intervention” OR “exercises training” OR “exercise training” OR "physical activity" OR "exercise")

A total of 41 articles
