# Supplementary material for: Effect of exercise training on blood pressure variability in adults: A systematic review and meta-analysis
Source: PLoS One. 2023 Oct 18;18(10):e0292020. doi: 10.1371/journal.pone.0292020 (PMC10584136; doi:10.1371/journal.pone.0292020)
Supplement: S3 File — (PDF) [file pone.0292020.s004.pdf]

Electronic Supplementary Material Appendix S2

Quality of studies – Grids

SIGN Methodology Checklist 2: Controlled Trials

Study identification (*Include author, title, year of publication, journal title, pages*)

Guideline topic:	Key Question No:	Reviewer:

Before completing this checklist, consider:

1. Is the paper a **randomised controlled trial** or a **controlled clinical trial**? If in doubt, check the study design algorithm available from SIGN and make sure you have the correct checklist. If it is a **controlled clinical trial** questions 1.2, 1.3, and 1.4 are not relevant, and the study cannot be rated higher than 1+
2. Is the paper relevant to key question? Analyse using PICO (Patient or Population Intervention Comparison Outcome). IF NO REJECT (give reason below). IF YES complete the checklist.

Reason for rejection: 1. Paper not relevant to key question ☐ 2. Other reason ☐ (please specify):

SECTION 1: INTERNAL VALIDITY

	In a well conducted RCT study…	***Does this study do it?***
1.1	The study addresses an appropriate and clearly focused question.	Yes ☐ No ☐<br>Can't say ☐
1.2	The assignment of subjects to treatment groups is randomised.	Yes ☐ No ☐<br>Can't say ☐
1.3	An adequate concealment method is used.	Yes ☐ No ☐<br>Can't say ☐
1.4	The design keeps subjects and investigators 'blind' about treatment allocation.	Yes ☐ No ☐<br>Can't say ☐
1.5	The treatment and control groups are similar at the start of the trial.	Yes ☐ No ☐<br>Can't say ☐
1.6	The only difference between groups is the treatment under investigation.	Yes ☐ No ☐<br>Can't say ☐
1.7	All relevant outcomes are measured in a standard, valid and reliable way.	Yes ☐ No ☐<br>Can't say ☐
1.8	What percentage of the individuals or clusters recruited into each treatment arm of the study dropped out before the study was completed?	
1.9	All the subjects are analysed in the groups to which they were randomly allocated (often referred to as intention to treat analysis).	Yes ☐ No ☐<br>Can't say ☐ Does not apply ☐
1.10	Where the study is carried out at more than one site, results are comparable for all sites.	Yes ☐ No ☐<br>Can't say ☐ Does not apply ☐

SECTION 2: OVERALL ASSESSMENT OF THE STUDY		
2.1	How well was the study done to minimise bias? *Code as follows:*	High quality (++)☐<br>Acceptable (+)☐<br>Low quality (-)☐<br>Unacceptable – reject 0 ☐
2.2	Taking into account clinical considerations, your evaluation of the methodology used, and the statistical power of the study, are you certain that the overall effect is due to the study intervention?	
2.3	Are the results of this study directly applicable to the patient group targeted by this guideline?	
2.4	**Notes.** Summarise the authors' conclusions. Add any comments on your own assessment of the study, and the extent to which it answers your question and mention any areas of uncertainty raised above.	
