# Supplementary material for: Effect of exercise training on blood pressure variability in adults: A systematic review and meta-analysis
Source: PLoS One. 2023 Oct 18;18(10):e0292020. doi: 10.1371/journal.pone.0292020 (PMC10584136; doi:10.1371/journal.pone.0292020)
Supplement: S4 File — (PDF) [file pone.0292020.s005.pdf]

SIGN assessment of 11 studies											
study/assess item	Q1	Q2	Q3	Q4	Q5	Q6	Q7	Q8	Q9	Q10	yes%
Diaz,2012	1	-	-	-	1	1	1	1	0	0	50
Katrina,2019	1	1	1	0	1	1	1	1	0	0	70
Mariano，2020	1	-	-	-	1	1	1	0	0	0	40
Betolaza， 2020	1	1	1	1	1	1	1	1	0	0	80
Caminiti， 2021	1	-	-	-	1	1	1	1	0	0	50
Chehuen,2021	1	1	1	0	1	1	1	1	0	0	70
Jamie,2021	1	1	1	0	1	1	1	1	0	0	70
Seidel， 2021	1	1	1	0	1	1	1	1	0	0	70
Baross, 2021	1	1	1	0	1	1	1	1	0	0	70
Batista，2022	1	-	-	-	1	1	1	1	0	0	50
Caminiti， 2022	1	-	-	-	1	1	1	1	0	0	50

SIGN assessment of 6 RCTs											
study/assess item	Q1	Q2	Q3	Q4	Q5	Q6	Q7	Q8	Q9	Q10	yes%
Katrina,2019	1	1	1	0	1	1	1	1	0	0	70
Betolaza， 2020	1	1	1	1	1	1	1	1	0	0	80
Chehuen,2021	1	1	1	0	1	1	1	1	0	0	70
Jamie,2021	1	1	1	0	1	1	1	1	0	0	70
Seidel， 2021	1	1	1	0	1	1	1	1	0	0	70
Baross, 2021	1	1	1	0	1	1	1	1	0	0	70
