# Supplementary figures and images for: Effect of exercise training on blood pressure variability in adults: A systematic review and meta-analysis
Source: PLoS One. 2023 Oct 18;18(10):e0292020. doi: 10.1371/journal.pone.0292020 (PMC10584136; doi:10.1371/journal.pone.0292020)

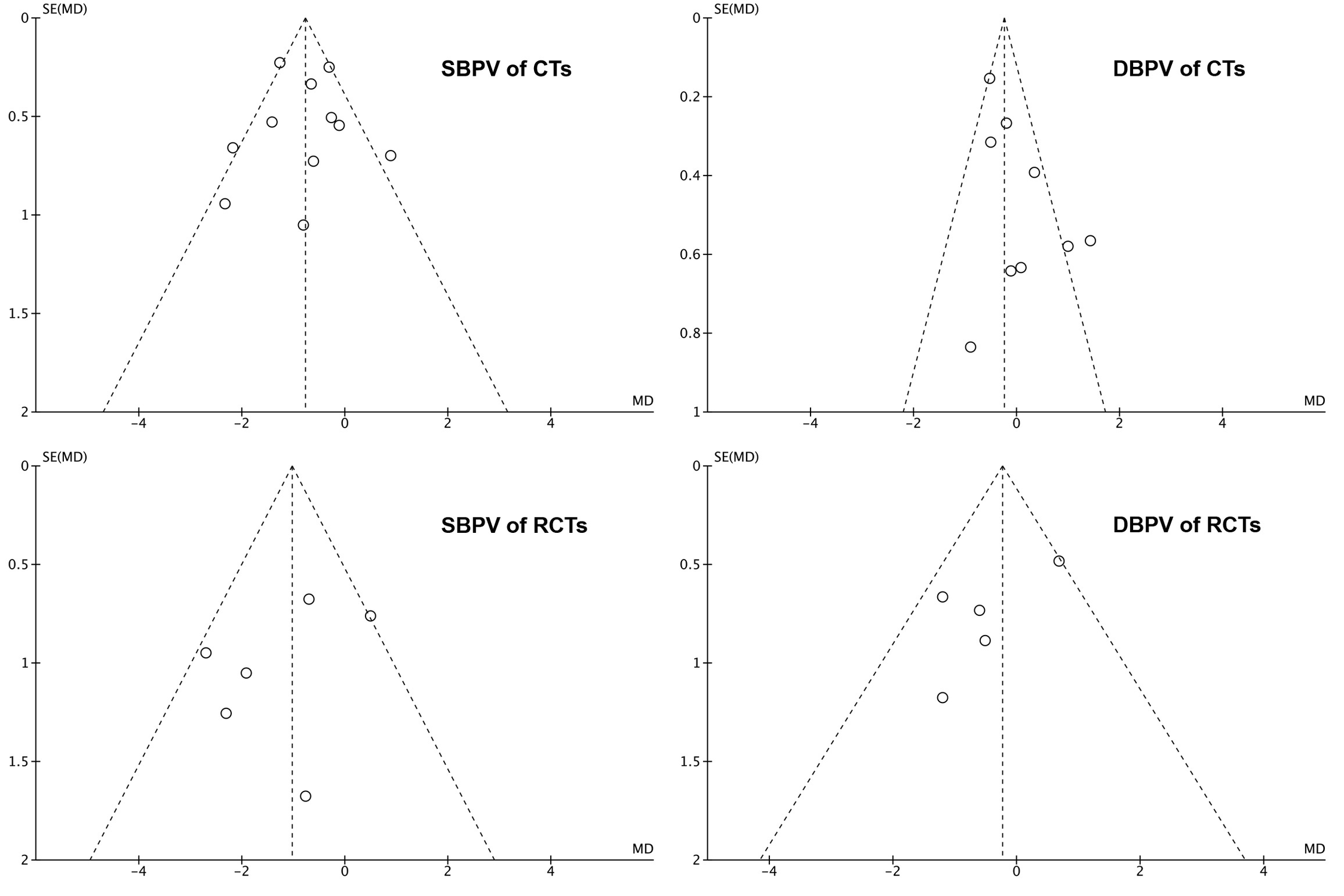

Supplement: S5 File — (TIF) [file pone.0292020.s006.tif]
